# Coupling between the lever arm and active site via an N-terminal extension tunes force sensitivity in the myosin-1 family

**DOI:** 10.1073/pnas.2502977122

**Published:** 2025-04-07

**Authors:** Harry W. Rathbone, Anne Houdusse

**Affiliations:** ^a^Structural Motility, Institut Curie, Université Paris Sciences et Lettres, Sorbonne Université, CNRS UMR144, Paris 75248, France

Myosins are ubiquitous eukaryotic molecular motors responsible for providing tensile forces in cells and motile power at the nanoscale. This allosteric mechanoenzyme undergoes a series of conformational changes associated with adenosine triphosphate (ATP) hydrolysis and actin binding, aka the motor cycle, which result in the force-generating swing of a lever arm (the powerstroke). Evolution has diversified the basic myosin structure thus altering the structural progression between each conformation for use in various cellular niches. The myosin motor cycle consists of concurrent chemical and mechanical transitions. When attached to actin, hydrolyzed ATP products, adenosine diphosphate (ADP) and phosphate, are successively released in parallel to structural changes leading to a lever arm swing producing force. Once all products are released, ATP can re-enter the active site causing detachment from actin, resetting the cycle (1). The immense subtlety of structural movement inherent in allosteric motors is well appreciated but little understood.

The rate of transitions between the conformations of the myosin motor are dependent on a large number of factors with one of the fascinating aspects of myosin motor function being their response to load. This force sensing can occur at different stages in the motor cycle. Some myosins release from actin under relatively low loads and others can sustain actin binding with increased load, acting as an anchor. Diversity in load-sensitive kinetics tune their cellular function (2). An example of this comes from the myosin-1 family which generates a mechanical connection between the actin cortex and the cell membrane operating as an ensemble of monomeric motors to create traction, force sensing, and anchorage (2, 3). Myo1c is of particular interest as it contributes to asymmetric morphogenesis of organs hinging on cellular-level chirality and linked to a peculiarity seen in gliding assays where Myo1c induces a distinct leftward rotation of actin filaments (4, 5).

Chavali et al. (6) describe with atomic detail how the mechanical transitions performed by Myo1c lead to its unique biophysical properties. Three actin-bound structural states from Mg^2+^.ADP bound, ADP bound intermediate to Rigor (nucleotide-free) are determined, revealing conformations explored during the swing of the lever arm of the powerstroke. These provide unique insights into how force sensitivity and mechanical output can be varied and explain how myosins have evolved to serve distinct cellular functions.

The majority of myosins sense force during their ADP release transition. Myo1b is one such myosin and its ADP release is slowed ~100-fold by mechanical loads of <1 pN, forming a catch bond and making it a particularly effective tension-sensitive anchor in this range (7). On the other hand, Myo1c is relatively insensitive to load, performing a slow powerstroke that becomes load sensitive beyond 1 pN (8). Moreover, applied load slows the binding of ATP in Myo1c, where ATP binding is required to release the motor from actin in turn resetting the motor cycle. In contrast to Myo1b, Myo1c is thus suited to generate power under load and can act as a slow transporter performing a powerstroke even when load reaches 4 pN. Force sensing is known to be, at least in part, determined by the N-Terminal Extension (NTE) in myosin-1. Tuning of ADP release by an NTE has also been reported in PfMyoA (9). In that case, the phosphorylation of a serine in the NTE is sufficient to speed up ADP release due to its effect in promoting the swing of the lever arm. Understanding how molecular motors with similar unloaded ATP kinetics can adopt drastically different loaded kinetics and mechanics remains a mystery.

**Fig. 1.**
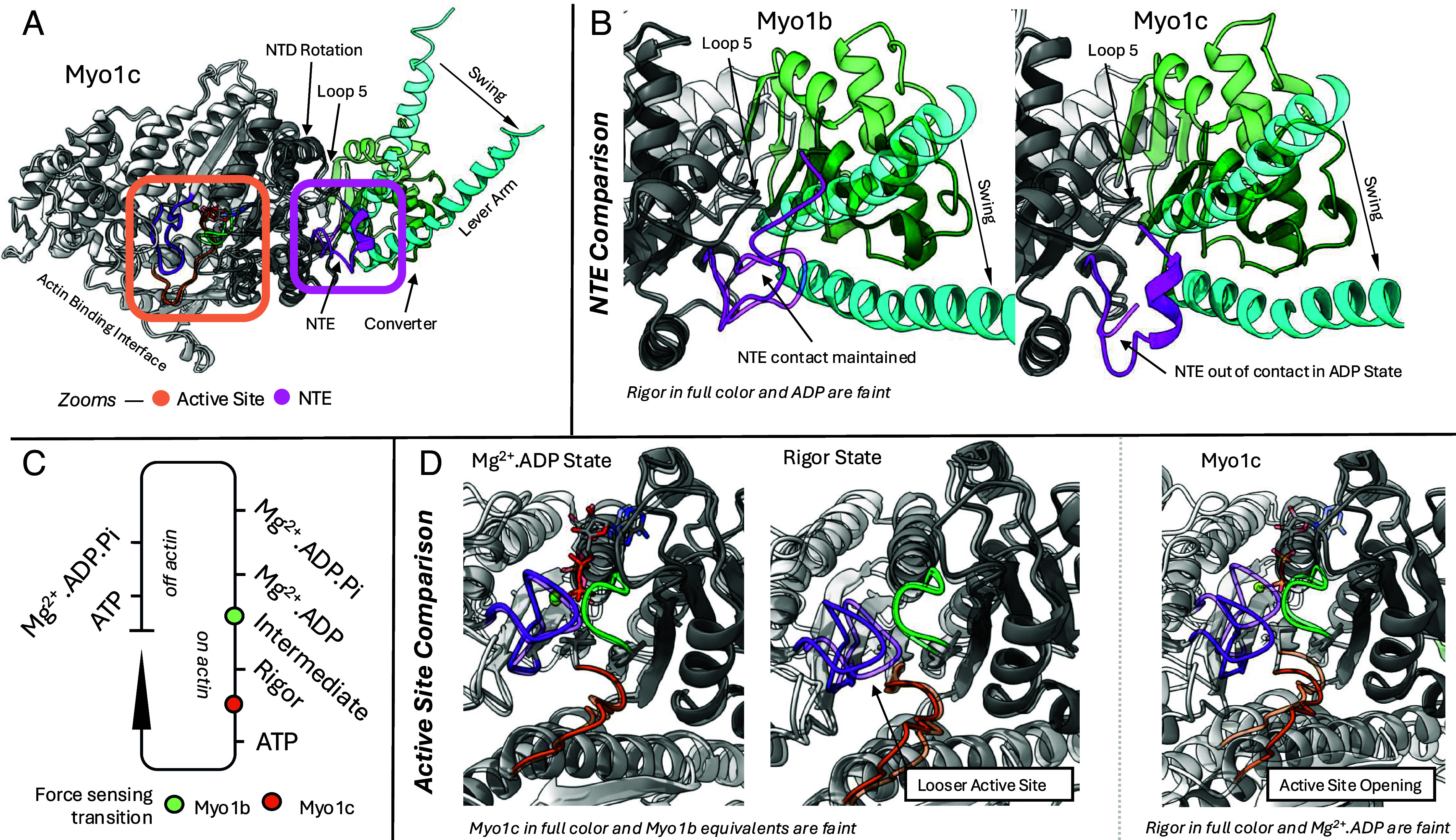
Comparison of structural transitions between Myo1b (force sensitive during ADP release) and Myo1c (force sensitive during ATP binding). (*A*) Overview of key regions of study of the myosin motor including the conformational changes during ADP release. (*B*) Comparisons of the NTE binding between Myo1b and Myo1c. Note that the Rigor state is in full color and the Mg^2+^.ADP states are in faint coloring. (*C*) Diagram of the motor cycle including steps which are force sensitive. (*D*) Comparisons of the changes of the active site of Myo1c and Myo1b in either the Mg^2+^.ADP or Rigor state (*Left* and *Center*) or comparison of the active site movements for Myo1c during ADP release (*Right*). Note that in the *Left* and *Center*, Myo1c is in full color and Myo1b is faint and on the *Right* the Rigor state is in full color and the Mg^2+^.ADP states are in faint coloring.

Using high-resolution cryogenic electron microscopy (cryo-EM), Chavali et al. (6) now provide a detailed structural basis for the difference in force sensitivity observed between Myo1b and Myo1c. They propose that load sensing requires strong coupling between the lever arm and the active site, which occurs in Myo1b but not Myo1c. In Myo1b, coupling occurs during the conformational change from Mg^2+^.ADP to Rigor through a coordination of the NTE and adjacent Loop-5 (Fig. 1) which interact with the lever arm. This coupling is seen in all the actin-bound states determined (Mg^2+^.ADP bound, ADP bound intermediate, and Rigor/nucleotide free). Release of Mg^2+^ and ADP in Myo1b requires the full lever arm swing to Rigor to open the active site completely and is strongly inhibited by load. The NTE is essential for this inhibition as it maintains coupling of the active site with the lever arm, thereby sensing force and preventing the swing and the release of Mg^2+^ and ADP (10). In contrast, in Myo1c, the NTE is unable to perform this role as it is not part of the initial interface (Fig. 1). The lever arm can thus swing independently of load while the active site opens to promote ADP release. The Myo1c NTE docks at the end of the powerstroke adopting a helical conformation stabilizing interactions across the lever arm, NTE, and N-terminal subdomain (Fig. 1). The nucleotide binding site in the Rigor (pre-ATP binding) state of Myo1c differs from that of Myo1b. A hydrogen bonding network is disrupted consistent with reduced nucleotide binding (Fig. 1). The authors further show that deletion of the NTE in Myo1c enables the formation of this network as in Myo1b. This is consistent with kinetic studies describing fast ATP binding and slow release of ADP of this truncated construct (11). The Myo1c structures reveal why the lever arm swing and ADP release are less sensitive to load: The transitions that open the active site differ significantly from Myo1b (Fig. 1) and are not directly coupled to the lever arm swing. The effect of sequence alterations that distinguish these two mechanoenzymes could not be divined ab initio without structure determination, particularly in the case of the intrinsically disordered NTE as it docks with the motor domain.

Myo1c presents another interesting puzzle to researchers in that it turns actin leftward during its lever arm swing (4). Chavali et al. (6) describe the structural determinants of this behavior as a series of compounded and amplified structural shifts. As a result, there is an approximately threefold increase in skew of the lever arm swing (32°) of Myo1c off the actin axis compared to Myo1b. The way that myosin sits on actin, its perch, is the first structural difference. Myo1c is perched on actin with a rotation of ~7° compared to Myo1b. Such a change in perch is not uncommon and has been observed for other myosins with concomitant changes in peripheral myosin loops binding to actin (12). The authors show that even if the myosin were repositioned to the perch of Myo1b, the off-axis skew remains at 28°. A key change appears to be in the orientation of the lever arm in Rigor due to loss of contacts with Loop-5 and establishment of strong interactions with the NTE. Using these results and published crystal structures, the authors then modeled the full powerstroke using the complete protein. From this model, they show that the lever position at the end of the swing is nearly perpendicular to actin. The lever swing also has an off-axis component leading to possible torque (4), which is consistent with the role of the motor in breaking of cellular symmetry in vivo (5). Interestingly, the off-axis component of the lever arm in Rigor suggests a portion of the load is not aligned with the swing orientation. This might attenuate the reversal of the Myo1c stroke, increasing the lifetime of the actin-bound state in the presence of load >1 pN.

Chavali et al. (6) provide insight with structural detail into how the opening of the nucleotide binding site responds to and is directed by interactions with NTEs of myosin-1 and thus how force sensing occurs. This work also details how compounding allosteric structural alterations can dictate the direction of force generated at the motor domain. While discrete structural states provide crucial snapshots of possible states traversed by these myosins allowing for the insights above, the reality of molecular motors is much more complex and often underappreciated. Rather than a sequence of rigid states, the motor cycle consists of conformational ensembles in equilibrium with one another as the motor “jiggles” through a landscape of states comprising the mechanochemical cycle. One could thus imagine how load and the induced strain restrict the available conformational region for a given chemical state thus limiting the possibilities of passing between states and slowing or preventing transitions. This is also made manifest through the constraints imposed on the conformational exploration of the lever arm by the NTE. Understanding how distinct molecular motors sense load has far-reaching consequences. Load sensing in processive motors may be key to their progression due to the coupling of the leading and rear heads (13–15) and it is currently unknown how Myo6 motors can switch function from processive transporters to molecular anchors (16, 17). By way of analogy, similar effects are evident in the modulation of force production by Omecamtiv Mecarbil [a myotrope in stage 3 clinical trials with the potential for heart failure treatment (18)]. In this case, binding of the drug provides way of increasing the stiffness of bonds between the motor domain and the lever arm, mimicking the role described for the NTE and increasing the lifetime of the actomyosin bound states of cardiac myosin. Understanding how nature controls the tight interactions between the motor domain and the lever arm may open doors to new therapies.

Chavali et al. (6) describe with atomic detail how the mechanical transitions performed by Myo1c lead to its unique biophysical properties.

Continued technical advances will decipher how myosin mechanoenzymes can be tuned by load, mutation, or post-translational modifications. The nature of how load impacts the conformational landscape, compliance, and thus kinetics, will be defined through loaded dynamics and biophysical assays (19) in conjunction with structural insights into the conformational states captured while myosin is under strain. This will lead to a full mechanistic understanding of how force sensing controls the dynamics of the cytoskeleton in various essential cellular processes.
